# Fibrinogen Deficiency and Dysfunction in Postpartum Hemorrhage: Evidence, Thresholds, and Management Across Resource Settings

**DOI:** 10.3390/jcm15197565

**Published:** 2026-09-29

**Authors:** Gulshat Bilyalova, Aida Baibusunova, Assel Boshanova, Galym Shegenov, Bahit Anapina, Damir Biktashev, Kulyash Jaxalykova, Aigul Turgumbaeva, Assel Baubekova, Zhansulu Akylzhanova, Saltanat Kulbayeva, Zhanar Kozhakhmetova, Dinara Turzhanova, Ruslan Alexeyev

**Affiliations:** 1 Department of Obstetrics and Gynecology No. 2, Astana Medical University, 010000 Astana, Kazakhstan; bilyalova@ca-amu.kz (G.B.);; 2 Department of Obstetrics and Gynecology No. 1, Astana Medical University, 010000 Astana, Kazakhstan; 3 Department of Internal Diseases No. 2, Astana Medical University, 010000 Astana, Kazakhstan; 4 Department of Neonatology, Astana Medical University, 010000 Astana, Kazakhstan; 5 Department of Obstetrics and Gynecology, South Kazakhstan Medical Academy, 160000 Shymkent, Kazakhstan; 6 Scientific Research Institute of Radiology Named After Zh. H. Khamzabayev, Astana Medical University, 010000 Astana, Kazakhstan; 7 Department of Sports Medicine, Rehabilitation and Clinical Research, Kazakh National University of Sports, 010000 Astana, Kazakhstan; 8 Viamedis LLP, 010000 Astana, Kazakhstan

**Keywords:** pregnancy, postpartum hemorrhage, fibrinogen, hypofibrinogenemia, disseminated intravascular coagulation, placental abruption, amniotic fluid embolism, viscoelastic testing, cryoprecipitate, fibrinogen concentrate

## Abstract

Pregnancy is characterized by progressive hemostatic adaptation: increased fibrinogen synthesis, enhanced thrombin generation, and reduced fibrinolytic potential together prepare the maternal circulation for delivery-related blood loss. A fall in fibrinogen during pregnancy or the immediate postpartum period therefore does not carry the same meaning as an equivalent value outside of pregnancy. It may instead represent loss of a pregnancy-specific hemostatic reserve. Fibrinogen concentration is consistently associated with the severity of postpartum hemorrhage, but randomized trials have not shown that empirical replacement in all women with early postpartum hemorrhage improves clinical outcomes. This narrative review summarizes current evidence on pregnancy-associated fibrinogen deficiency and dysfunction, with emphasis on pathophysiology, trial findings, fibrinogen thresholds, replacement strategies, congenital fibrinogen disorders, and research gaps. Current data support using fibrinogen as both a severity marker and a selective treatment target, interpreted according to gestational age, bleeding phenotype, placental involvement, and resource setting. Direct evidence generated in low-resource settings is limited; no randomized trial of fibrinogen measurement or replacement has been conducted in such a setting.

## 1. Introduction

Obstetric hemorrhage remains the leading cause of maternal death worldwide [1,2]. Of coagulation factors, fibrinogen falls to a critically low concentration earliest during major hemorrhage, before the prothrombin time or activated partial thromboplastin time become abnormal [3,4]. Low fibrinogen during postpartum hemorrhage (PPH) predicts progression to severe bleeding and transfusion [3,5,6].

Fibrinogen is among the strongest early predictors of severe PPH, yet randomized trials of routine fibrinogen concentrate in early PPH have not improved outcomes [7,8,9]. Hypofibrinogenemia in pregnancy can arise from ongoing intravascular coagulation, accelerated fibrinolysis, dilution, impaired hepatic synthesis, or qualitative fibrinogen dysfunction [10,11,12]. Distinguishing among these mechanisms, not the concentration alone, is central to interpreting a single fibrinogen value.

Low-resource settings compound the problem. Delayed recognition of blood loss, limited staffing and transport, a high prevalence of antenatal anemia, unreliable laboratory access, blood component shortages, and cold chain interruptions all narrow the margin for error. This review sets out the molecular and mechanical pathways that determine fibrinogen concentration, function, and clot quality in obstetric hemorrhage. It then translates that evidence into a practical approach to recognition, assessment, treatment, surveillance, team coordination, and referral under resource constraints.

### Terms Used in This Review

“Low-resource,” “limited-resource,” and “small facility” are used inconsistently across the PPH literature. A facility without a laboratory and a facility with a laboratory but no viscoelastic testing face different decisions. We anchor our recommendations instead to the basic and comprehensive emergency obstetric and newborn care (BEmONC/CEmONC) framework. This is the classification most widely used in obstetric health systems research, and it stratifies facilities by function [13]. We extend this classification with the fibrinogen-specific capability at each tier:

- Tier 1 (BEmONC-level): no on-site blood transfusion and no rapid fibrinogen testing. Fibrinogen-directed therapy is not deliverable on site by definition; management is limited to the first-response bundle, uterotonics, tranexamic acid, a non-pneumatic anti-shock garment where available, and referral.
- Tier 2 (CEmONC-level without viscoelastic testing or concentrate): on-site blood transfusion and a standard clinical laboratory capable of Clauss fibrinogen, but no ROTEM/TEG and no fibrinogen concentrate. Cryoprecipitate or fresh frozen plasma are the available fibrinogen-rich products.
- Tier 3 (CEmONC-level with point-of-care testing): viscoelastic testing (ROTEM or TEG) and fibrinogen concentrate are stocked and available within the timeframe of active hemorrhage.

## 2. Methods

We searched PubMed/MEDLINE, Embase, and the Cochrane Central Register of Controlled Trials from database inception to 31 May 2026, with a final update on 15 July 2026. Search terms combined controlled vocabulary and free-text words for fibrinogen (fibrinogen, hypofibrinogenemia, afibrinogenemia, dysfibrinogenemia, FIBTEM, and viscoelastic), obstetric hemorrhage (postpartum hemorrhage, postpartum hemorrhage, obstetric hemorrhage, placental abruption, amniotic fluid embolism, and disseminated intravascular coagulation) and pregnancy (pregnancy, peripartum, puerperium, and maternal). No language restriction applied at the search stage. Non-English records that could not be assessed in full text were excluded at screening.

We included randomized controlled trials, prospective and retrospective cohort studies, case series informative for rare conditions (congenital fibrinogen disorders, amniotic fluid embolism), and guidelines from the WHO, FIGO, RCOG, ACOG, NATA, and ISTH. We excluded single case reports and non-peer-reviewed sources.

## 3. Obstetric Hemorrhage: Definitions, Timing, and Diagnosis

### 3.1. Definitions

Postpartum hemorrhage was traditionally defined as blood loss exceeding 500 mL after vaginal birth or 1000 mL after cesarean birth. The American College of Obstetricians and Gynecologists now defines it as cumulative blood loss of 1000 mL or more, or blood loss with signs or symptoms of hypovolemia, within 24 h of birth. This includes intrapartum loss and applies regardless of delivery route. Blood loss above 500 mL after vaginal birth remains abnormal and should prompt evaluation [14]. The Royal College of Obstetricians and Gynaecologists grades PPH as minor (500–1000 mL) or major (above 1000 mL); major bleeding subdivides into moderate (1000–2000 mL) and severe (above 2000 mL) [15]. Antepartum hemorrhage is bleeding from or into the genital tract from 24 + 0 weeks of gestation until birth [16].

The 2025 WHO guideline shifts emphasis from definition to action. It recommends first-response treatment once objectively measured blood loss reaches at least 500 mL, or at least 300 mL together with any abnormal hemodynamic sign, whichever occurs first within 24 h of birth. Abnormal signs include a pulse above 100 beats/min, a shock index above 1, a systolic blood pressure below 100 mmHg, or a diastolic blood pressure below 60 mmHg [17]. Vigilance should be greatest in the first 2 h.

### 3.2. Timing of Bleeding

Antepartum hemorrhage from 24 weeks until birth is dominated by placental abruption and placenta previa. Abruption is the commonest cause of low fibrinogen before delivery. Intrapartum and peripartum bleeding includes uterine rupture, abnormally invasive placenta, and amniotic fluid embolism, which is the classic cause of profound hypofibrinogenemia appearing before, or out of proportion to, visible blood loss. Primary PPH occurs within 24 h of birth. Causes are conventionally grouped as tone, trauma, tissue, and thrombin, with uterine atony accounting for most cases [1,17,18]. Secondary PPH, from 24 h to 12 weeks postpartum, most often reflects retained products of conception, endometritis, or subinvolution of the placental bed.

In a UK national population-based cohort, fibrinogen below 2 g/L during pregnancy or the immediate postpartum period occurred at a rate of approximately 1.7 per 10,000 maternities. About 60% of cases followed PPH caused by abruption, atony, or trauma. Amniotic fluid embolism and non-abruption placental causes carried the highest blood losses and the lowest fibrinogen concentrations. Eight percent of affected women had no hemorrhage at all [19]. No population-based study of equivalent design has yet been conducted in a low- or middle-income setting.

### 3.3. Objective Diagnosis and Measurement of Blood Loss

Visual estimation systematically underestimates blood loss and delays treatment. The E-MOTIVE cluster-randomized trial tested an alternative: a calibrated drape for objective measurement, combined with a first-response bundle of uterine massage, oxytocic drugs, tranexamic acid, intravenous fluids, genital tract examination, and escalation. The trial enrolled 210,132 women delivering vaginally across 80 secondary-level hospitals in Kenya, Nigeria, South Africa, and Tanzania. Severe PPH, laparotomy for bleeding, or death from bleeding occurred in 1.6% of the intervention group versus 4.3% with usual care (risk ratio 0.40, 95% CI 0.32–0.50). Hemorrhage detection rose from 51.1% to 93.1% [20]. E-MOTIVE is important evidence, generated directly in African settings, for objective blood loss assessment combined with prompt bundled treatment. The observed benefit reflects the bundle and its implementation, not any single component, including the drape [17,20,21].

Where quantification is not possible, the shock index (heart rate divided by systolic blood pressure) is a simple bedside adjunct. Values at or above approximately 0.9–1.0 predict transfusion requirement and adverse maternal outcome and have been proposed as a referral trigger where resources are limited [22].

## 4. Physiological Fibrinogen Adaptation in Pregnancy

Fibrinogen, factor VIII, von Willebrand factor, and the vitamin K-dependent factors VII, IX, and X all increase in normal pregnancy. Free protein S decreases, and fibrinolysis is attenuated through endothelial PAI-1 and placental PAI-2 [10,11,23,24]. Thrombin generation rises from the earliest weeks of the first trimester [25,26]. These adaptations reduce blood loss at placental separation. But they also mean that a fibrinogen concentration considered acceptable outside pregnancy can represent a substantial reduction at term.

Fibrinogen values in late pregnancy run substantially higher than nonpregnant values. Reference intervals do vary with population, assay, reagent, and analyzer. Szecsi et al. reported pregnancy-specific hemostatic reference intervals in 801 women. Siennicka et al. reported trimester-specific fibrinogen ranges of 2.60–6.56, 3.40–8.53, and 3.63–9.14 g/L for the first, second, and third trimesters, respectively [27,28]. A concentration of 2.5–3.0 g/L is unremarkable in a nonpregnant adult. At term, the same value can represent a fall of roughly half from baseline.

Throughout this review, 2 g/L is treated as a guideline-endorsed action threshold. No mechanistic study demonstrates a step-change in clot behavior precisely at this level. The threshold instead marks where cohort studies place the inflection in bleeding risk, and where guideline committees have set a pragmatic, testable target [17]. Normal third-trimester fibrinogen runs roughly double the nonpregnant upper-normal range. A value of 2 g/L is uninformative outside pregnancy, yet it can represent a fall of 50% or more from a woman’s own antenatal baseline.

One question remains unresolved: is the pregnancy fibrinogen reserve itself required for hemostasis, or does it act as a buffer that can be drawn down before function is compromised? Available evidence favors the buffer explanation. OBS2 did not improve outcomes in its overall randomized population. Pre-specified analyses suggested replacement was unlikely to help when FIBTEM A5 exceeded 12 mm or Clauss fibrinogen exceeded 2 g/L. Benefit below those levels stayed uncertain, because too few enrolled women had a concentration that low [9]. During active bleeding, a decline in fibrinogen tracks worsening hemorrhage, but its magnitude depends on cause, baseline concentration, timing, resuscitation, and assay. Absent overt bleeding, an unexpected decline may warrant evaluation for pathological causes.

## 5. Acquired Fibrinogen Deficiency and Dysfunction in Obstetric Hemorrhage: Mechanisms

**Figure 1:**
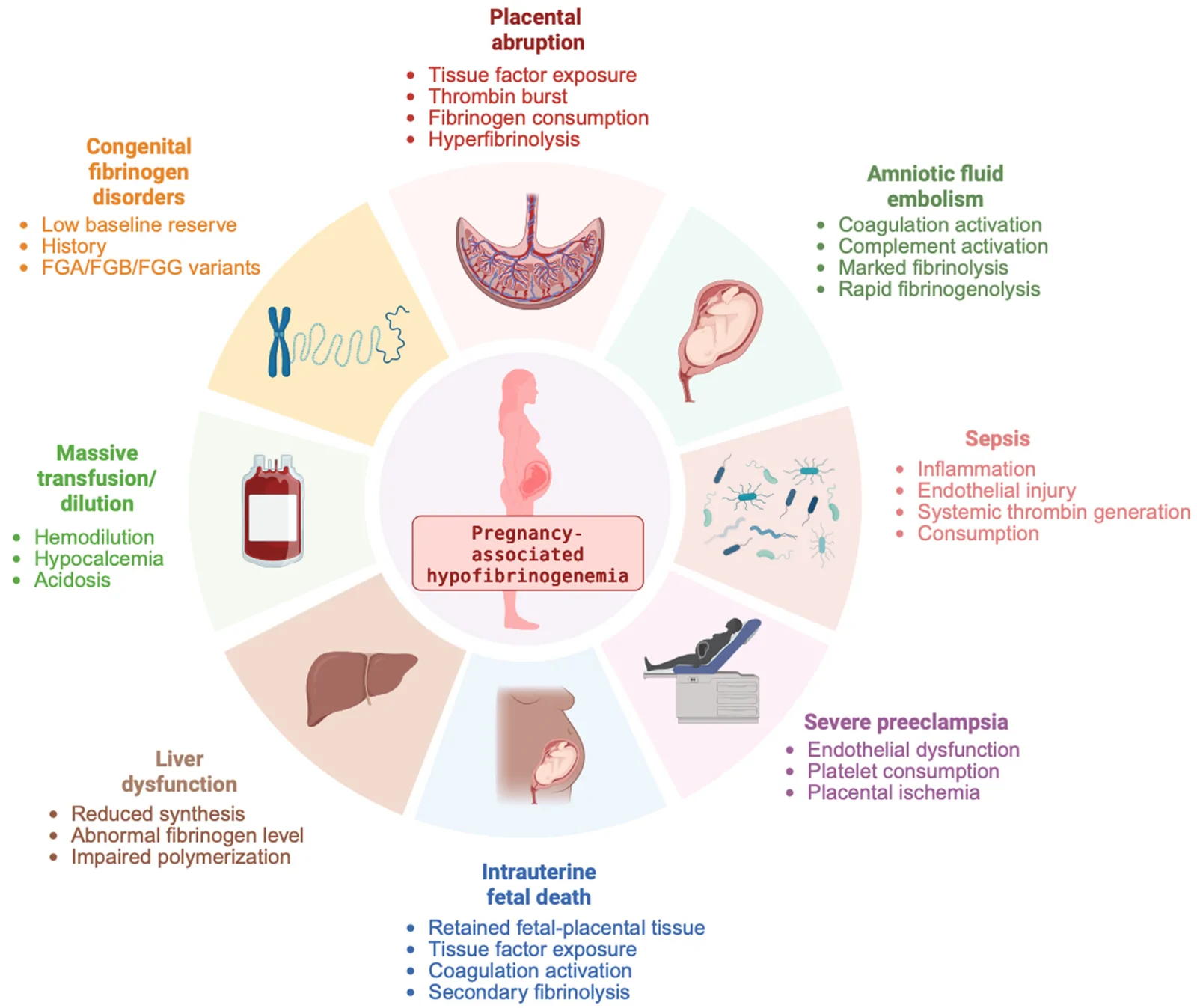
Causes and mechanisms of pregnancy-associated hypofibrinogenemia. Fibrinogen concentration falls through consumption following coagulation activation, degradation by plasmin, loss and dilution, and reduced hepatic synthesis.

Five mechanisms account for most pregnancy-associated hypofibrinogenemia and commonly act in combination (Figure 1).

Consumption following coagulation activation is the dominant and fastest mechanism. Tissue factor, exposed by injured or separated placental tissue, binds factor VIIa and activates factors X and IX. Amplified on activated platelet surfaces, this triggers a thrombin burst. Thrombin cleaves fibrinopeptides A and B from circulating fibrinogen, and the resulting fibrin monomers polymerize within the retroplacental space and, once activation becomes systemic, throughout the microcirculation. Fibrinogen is removed from plasma as it converts to fibrin, and platelets, factor XIII, and natural anticoagulants are consumed alongside it. When generalized, this constitutes disseminated intravascular coagulation (DIC) [29,30,31,32,33,34].

Plasmin acts on both formed fibrin and circulating fibrinogen. Thrombin generation and tissue injury provoke endothelial release of tissue plasminogen activator. Decidua and amniotic fluid supply additional plasminogen activators. Placental separation removes the major placental source of PAI-2, and endothelial PAI-1 regulation may also be disturbed during hemorrhage and shock [30,35,36]. Fibrin and fibrinogen degradation products further interfere with polymerization, so the clot formed from any remaining fibrinogen is weaker than its concentration suggests.

Dilution takes longer to reach a critical threshold in pregnancy because baseline fibrinogen is high. Obstetric practice applies a correspondingly higher treatment threshold, and fibrinogen still reaches critical concentrations before other factors do [4]. Factor XIII and platelets dilute and consume in parallel. Ionized calcium falls through citrate chelation from transfused components. Hypothermia, acidemia, and hypocalcemia compound the defect further, by slowing enzymatic coagulation and impairing polymerization. The Clauss assay runs at 37 °C and does not capture these effects, so the reported concentration can overstate true hemostatic capacity [17,35,37,38,39,40,41,42,43,44]. This mechanism dominates atony-dominant and traumatic hemorrhage, where the primary defect is mechanical. It is largely preventable through crystalloid restraint and early definitive hemostasis.

Acute fatty liver of pregnancy, pre-existing liver disease, and HELLP syndrome all reduce production. They can also yield post-translationally abnormal fibrinogen—an acquired dysfibrinogenemia in which functional activity is disproportionately reduced relative to antigen [42]. Fibrinogen is typically preserved in uncomplicated HELLP. A low concentration there should prompt a search for concealed abruption, DIC, or acute fatty liver.

Fibrinogen function can finally be impaired at a normal antigen concentration: in congenital dysfibrinogenemia, Clauss activity is low while immunologic fibrinogen is normal. This activity–antigen discordance is a characteristic clue, not a diagnosis by itself. Confirmation requires repeat testing, exclusion of analytical and acquired causes, and, where appropriate, specialist or genetic evaluation [45,46,47,48,49,50,51].

### Fibrinogen Concentration Versus Fibrinogen Function

Fibrinogen concentration and fibrinogen function are related but distinct properties, and a normal concentration does not guarantee a functionally adequate clot.

Concentration is how much fibrinogen protein is present in plasma, in g/L. It is measured by the Clauss assay or the fibrinogen antigen assay, through different methods. Clauss is a functional clotting time assay calibrated against a fibrinogen standard and is the test behind most obstetric thresholds. The antigen assay measures immunologic protein mass regardless of clotting activity. It becomes informative when congenital or acquired dysfibrinogenemia is suspected since Clauss–antigen discordance is the characteristic clue for that disorder group [46,47,50].

Function refers to whether the available fibrinogen can generate a mechanically adequate clot. This depends on steps downstream of concentration: thrombin-mediated cleavage of fibrinopeptides A and B; polymerization of the resulting fibrin monomers into protofibrils and then a three-dimensional network; factor XIII-mediated cross-linking that stabilizes the network; and platelet incorporation through glycoprotein IIb/IIIa binding to fibrin(ogen). Each step is independently modulated by pH, temperature, hematocrit, ionized calcium, and inflammatory change. The resulting clot is then subject to fibrinolysis, in which plasmin degrades both fibrin and fibrinogen. The degradation products themselves interfere with further polymerization [48,49,51]. Viscoelastic assays such as FIBTEM measure this functional output directly, as clot amplitude, rather than fibrinogen mass. They are better described as functional surrogates for fibrinogen than as alternative ways of measuring what the Clauss or antigen assay measures.

De Lloyd et al. found acute obstetric coagulopathy during PPH associated with hyperfibrinolysis and an acquired dysfibrinogenemia [35]. Ide et al. found significantly higher fibrin/fibrinogen degradation product and D-dimer concentrations below a Clauss fibrinogen of 0.5 g/L than above 1.0 g/L. This is consistent with degradation products adding a functional deficit at the lowest concentrations [30]. Clot firmness and hemostatic adequacy during active bleeding depend on both properties together. An approach that considers only numeric fibrinogen concentration, without clot quality, risks missing a clinically important functional deficit.

## 6. Causes of Obstetric Hemorrhage and Their Effect on Fibrinogen

### 6.1. Uterine Atony, Trauma, Retained Tissue, and Anatomic Bleeding

Uterine atony is the most common cause of primary PPH [1,17,18]. Genital tract lacerations, retained placental tissue, uterine inversion or rupture, placenta previa, placenta accreta spectrum, and surgical injury are additional major causes (Table 1).

Charbit et al. studied 128 women with PPH requiring prostaglandin infusion; 50 developed severe PPH. Fibrinogen measured early in bleeding was the only standard coagulation variable independently associated with progression to severe hemorrhage. Risk of severe PPH rose 2.63-fold per 1 g/L decrease in fibrinogen at enrollment (95% CI 1.66–4.16). A concentration at or below 2.0 g/L carried 100% positive predictive value for severe PPH. A concentration above 4.0 g/L carried a negative predictive value of 79% [3]. These predictive values come from a single cohort already receiving second-line uterotonic therapy and should not be adopted as universal diagnostic or treatment thresholds. Cortet et al. subsequently confirmed the association between lower fibrinogen and PPH severity in a prospective dataset [5].

Coleman et al. examined the mechanism directly in 81 women: 66 with PPH and 15 controls. Compared with the controls, women with PPH had lower fibrinogen, reduced peak and maximal-rate thrombin generation, and lower lysis at 30 min after maximal clot strength following tissue plasminogen activator challenge. This pattern indicates relative fibrinolytic resistance, rather than hyperfibrinolysis, in this cohort. They also had lower tissue factor and higher tissue factor pathway inhibitor levels [52].

### 6.2. Placental Abruption

Placental abruption begins with hemorrhage from maternal vessels in the decidua basalis, separation of the placenta from the uterine wall, and exposure of tissue factor-rich decidual and trophoblastic material.

Tikkanen summarized the epidemiology of placental abruption, reporting an incidence of approximately 0.4–1% of pregnancies, with major maternal and perinatal consequences [53]. Koroglu et al. reported a mean predelivery fibrinogen concentration of 2.21 ± 1.12 g/L in placental abruption. In multivariable analysis, fibrinogen independently predicted PPH and red cell and plasma transfusion. PPH risk rose below 1.3 g/L. Overt DIC and transfusion risk rose below 1.0 g/L; values below 2.5 g/L were associated with adverse neonatal outcomes [54].

Fibrinogen does not diagnose placental abruption, which remains a clinical and obstetric diagnosis. What matters clinically is not only the absolute concentration but the abrupt decline from pregnancy baseline (Table 2).

### 6.3. Amniotic Fluid Embolism

Amniotic fluid embolism (AFE) is uncommon, at approximately 2–8 cases per 100,000 deliveries depending on diagnostic criteria. It is nonetheless strongly associated with sudden cardiovascular collapse, respiratory failure, and coagulopathy [55,56]. Amniotic fluid contains tissue factor and other procoagulant material, demonstrated in early mechanistic work by Lockwood et al. [29]. Its hemostatic phenotype combines intense coagulation activation with pronounced fibrinolytic activation.

Mechanistically, Ide et al. compared 27 AFE cases from the Japanese AFE Registry, 12 severe abruption cases complicated by DIC, and 23 peripartum controls. Prothrombin fragment 1 + 2 and plasmin-alpha2-plasmin inhibitor complex were markedly increased in both AFE and severe abruption, versus the controls. Tissue plasminogen activator was significantly increased in AFE specifically and total thrombin-activatable fibrinolysis inhibitor (TAFI) decreased [30]. Tanaka et al. examined 46 maternal deaths related to AFE. Of the 15 with a fibrinogen result, the concentration was undetectable (below 0.50 g/L) and every case with blood loss of at least 1000 mL showed a low value [57]. Hasegawa et al. separately described eight maternal deaths from placental abruption. Where fibrinogen was measured, the initial value was below 1.0 g/L (100 mg/dL) in every case [58]. Ide’s registry cohort showed that blood loss at sampling was already substantial, with medians of 4900 mL in AFE and 2648 mL in severe abruption. This cohort therefore does not itself demonstrate hypofibrinogenemia preceding visible loss [30]. Complement activation, thrombin generation, tPA release, TAFI consumption, plasmin generation, and degradation product accumulation together produce a weak, rapidly degraded clot.

The lowest, most striking fibrinogen values come from case series with early sampling, while registry data with later sampling show substantial concurrent blood loss. Profound hypofibrinogenemia should therefore be anticipated in severe AFE but not presented as an early or diagnostic feature of AFE in general.

### 6.4. Sepsis, Hypertensive Disorders, Fetal Death, Liver Dysfunction, and Major Dilution

In sepsis, fibrinogen is an acute-phase protein as well as a coagulation substrate. A normal or increased early value therefore does not exclude evolving coagulopathy. Cytokine-driven tissue factor expression and endothelial anticoagulant loss produce microvascular fibrin deposition before overt bleeding, so hypofibrinogenemia usually signals advanced or overt DIC rather than early sepsis [31,32,33,34,59]. A low or rapidly falling fibrinogen in septic pregnancy is a severe warning sign, particularly alongside thrombocytopenia, prolonged PT/aPTT, or rising FDP/D-dimer levels. It should be trended serially, not read as a single value [31,32,33,34,60].

Severe preeclampsia and HELLP-spectrum disease primarily cause endothelial and microangiopathic injury. Fibrinogen is typically preserved until advanced disease, concomitant abruption, hepatic dysfunction, or overt DIC supervenes. A low value here should therefore prompt a search for one of these complications, not attribution to preeclampsia itself [12,31,32,39]. Intrauterine fetal death is not invariably associated with hypofibrinogenemia. Risk rises specifically when prolonged retention coexists with abruption, infection, or DIC. Severe hepatic failure reduces synthesis and can produce acquired dysfibrinogenemia. Major hemorrhage or transfusion may superimpose dilutional coagulopathy on any of these conditions.

**Table 1:** Causes and mechanisms of pregnancy-associated fibrinogen deficiency and dysfunction.

Clinical Context	Pathophysiological Process	Molecular Mechanism	References
Normal late pregnancy	Adaptive hypercoagulability; progressive fibrinogen rise; antifibrinolysis	Increased hepatic synthesis; higher thrombin generation; PAI-1/PAI-2 suppress tPA/uPA	[10,11,12,23,24,25,26,27,28]
Uterine atony-dominant PPH	Mechanical failure of compression; coagulopathy usually secondary to loss/dilution	Dilutional loss of fibrinogen, FXIII, calcium, platelets; acidosis/hypothermia impair polymerization	[1,3,5,6,7,8,35,37,38,41,43,44,52]
Placental abruption	Retroplacental bleeding; tissue-factor exposure; thrombin burst; consumption	Decidual tissue factor activates FXa; plasmin activation raises FDPs/D-dimer	[25,29,30,39,43,53,54,58]
Amniotic fluid embolism	Cardiorespiratory collapse with coagulation and marked fibrinolytic activation	Tissue factor exposure; complement activation; tPA rises, TAFI falls; fibrinogenolysis	[12,25,29,30,55,56,57]
Sepsis with overt DIC	Cytokine-driven coagulation activation; endothelial injury; late consumption	IL-1/IL-6/TNF-alpha induce tissue factor; thrombomodulin/protein C downregulated; PAI-1 suppresses fibrinolysis	[31,32,33,34,59,60]
Severe preeclampsia/HELLP	Endothelial dysfunction; platelet activation; DIC in severe cases only	sFlt-1-mediated endothelial injury; hepatic synthetic impairment in severe disease	[1,12,31,32]
Intrauterine fetal death	Retained tissue-factor-rich material; coagulation activation in selected cases	Thromboplastin-rich material activates tissue-factor pathway; secondary fibrinolysis	[1,31,32]
Massive transfusion/dilution	Hemodilution; hypothermia; acidosis; calcium depletion	Crystalloid/red cell dilution of fibrinogen and FXIII; impaired polymer assembly	[6,35,37,38,39,40,41,42,43,44]
Liver dysfunction/acquired dysfibrinogenemia	Reduced synthesis; abnormal fibrinogen in severe hepatic disease	Altered glycosylation impairs polymerization	[42,46,47,48,49]
Congenital afibrinogenemia/hypofibrinogenemia	Quantitative deficiency from impaired synthesis, assembly, or secretion	FGA/FGB/FGG variants impair chain production or hexamer assembly/secretion	[45,46,61,62,63,64,65,66,67,68,69,70,71]
Dysfibrinogenemia/hypodysfibrinogenemia	Qualitative dysfunction, with or without reduced antigen	FGA Arg35, FGG Arg301 hotspots; altered polymerization or fibrinolytic resistance	[45,46,47,48,49,50,51,71]

Abbreviations: DIC, disseminated intravascular coagulation; FDPs, fibrin/fibrinogen degradation products; FXIII, factor XIII; PAI, plasminogen activator inhibitor; PPH, postpartum hemorrhage; TAFI, thrombin-activatable fibrinolysis inhibitor; and tPA, tissue plasminogen activator.

**Table 2:** Clinical interpretation and management according to fibrinogen status.

Clinical Context	Typical Fibrinogen Trajectory and Key Data	Clinical Interpretation	References
Normal late pregnancy	Third-trimester range ~3.6–9.1 g/L, roughly double nonpregnant range	A value unremarkable outside pregnancy may reflect a large fall from personal baseline	[10,11,12,23,24,25,26,27,28]
Uterine atony-dominant PPH	Charbit et al.: 2.63-fold risk increase per 1 g/L fall; ≤2.0 g/L, PPV 100% for severe PPH; >4.0 g/L, NPV 79% *	Strong severity biomarker across its range; specific predictive values not generalizable as universal cut-offs	[3,5,6]
Placental abruption	Köroğlu et al.: mean 2.21 ± 1.12 g/L; PPH risk rises <1.3 g/L; DIC/transfusion risk <1.0 g/L; adverse neonatal outcome <2.5 g/L	Severity marker, DIC marker, and outcome correlate together rather than as independent signals; does not diagnose abruption	[25,53,54,58]
Amniotic fluid embolism	Fatal case series: <0.50 g/L in 14/15 (Tanaka); <1.0 g/L in all measured cases of 8 fatal abruptions (Hasegawa); registry data show high concurrent blood loss	Profound hypofibrinogenemia expected in severe AFE, but evidence is from selected severe/fatal cases	[30,55,56,57,58]
Sepsis with overt DIC	Normal/high early (acute-phase reactant); falls with progression to overt DIC	A normal early value does not exclude evolving coagulopathy	[31,32,33,34,60]
Severe preeclampsia/HELLP	Typically preserved until advanced disease	A low value is not explained by preeclampsia itself	[12,31,32,39]
Massive transfusion/dilution	Often among the first factors to reach critical levels; FIBTEM A5 thresholds vary by algorithm	Largely preventable with crystalloid restraint and early hemostasis	[6,35,37,38,39,40,41,42,43,44]
Congenital/acquired fibrinogen disorders	Diguisto et al.: <5% of 124 women with fibrinogen <2.0 g/L had a pre-existing inherited disorder	Uncommon cause overall; pregnancy may be the first unmasking event	[19,45,46,47,48,49,50,51,61,62,63,64,65,66,67,68,69,70,71]

* PPV/NPV from a single cohort already receiving second-line uterotonics, without a healthy control group. Abbreviations: DIC, disseminated intravascular coagulation; and PPH, postpartum hemorrhage.

## 7. Fibrinogen as a Severity Marker and as a Treatment Target

Low fibrinogen is among the strongest laboratory markers of deterioration in PPH, and is consistently associated with transfusion and invasive hemostatic intervention [3,5,6]. The association is biologically plausible: pregnancy begins with a high fibrinogen reserve, and major hemorrhage, consumption, and dilution reduce it. Five randomized trials have tested that proposition directly (Table 3).

In FIB-PPH, Wikkelso et al. randomized 249 women to a fixed 2 g dose of fibrinogen concentrate or placebo early in PPH. Mean blood loss at inclusion was 1459 mL and mean fibrinogen was 4.5 g/L; red cell transfusion to six weeks postpartum and secondary outcomes did not differ [7]. In FIDEL, Ducloy-Bouthors et al. randomized 437 women with persistent PPH after vaginal delivery, escalating from oxytocin to prostaglandin, to 3 g of fibrinogen concentrate or placebo. Mean blood loss was 877 mL and mean fibrinogen was 4.1 g/L. Early systematic administration did not improve the composite primary outcome, though it reduced hypofibrinogenemia and was safe [8].

OBS2 addressed the same question with point-of-care guidance. Collins et al. enrolled 663 women with PPH of 1000–1500 mL and randomized the 55 with FIBTEM A5 of 15 mm or less and continued bleeding to weight-adjusted fibrinogen concentrate or placebo. Concentrate did not reduce the primary outcome overall (adjusted incidence rate ratio for allogeneic units transfused, 0.72; 95% CI 0.3–1.7). But only seven of the 55 randomized women had Clauss fibrinogen at or below 2 g/L. Pre-specified analyses suggested replacement was unlikely to help above FIBTEM A5 12 mm or fibrinogen 2 g/L. Effect below those levels could not be determined [9]. A smaller pilot by Jokinen et al. randomized 60 women with PPH exceeding 1500 mL to a ROTEM-guided algorithm or conventional care. Red cell transfusion did not differ significantly (median 2 versus 3 units, *p* = 0.399), though plasma use and total blood loss were somewhat lower with ROTEM guidance. This trial tested an algorithm for overall coagulation factor support, rather than a fixed dose, and enrolled few women with severe hypofibrinogenemia [72].

Cryoprecipitate itself has been tested in only one trial. The UK ACROBAT pilot cluster-randomized trial assessed the feasibility of early cryoprecipitate delivery in severe PPH, defined as active bleeding within 24 h of birth requiring at least one red cell unit. Cryoprecipitate use was higher with intervention, 60% versus 31%. Red cell transfusion at 24 h was numerically lower (mean difference −0.6 units, 95% CI −1.2 to 0), and surgery or ICU admission was numerically lower too (odds ratio 0.6 and 0.4). The trial was a feasibility study, not powered for efficacy. Its authors concluded only that a full-scale trial appeared feasible [73].

Williams et al. pooled 12 studies: seven randomized and five non-randomized, 17,868 women in total. They rated the evidence on fibrinogen concentrate and cryoprecipitate as low-certainty and inconclusive for every patient-important outcome, including maternal death, severe morbidity, and thromboembolic events [74].

Across all five trials, and the Cochrane review pooling them [74], women with a documented fibrinogen at or below 2 g/L were rare. Only seven of 55 cases randomized in OBS2 met that threshold; most other trials enrolled none at all. These trials do not support routine empirical fibrinogen concentrate in unselected women with early PPH, most of whom had fibrinogen concentrations in or above the normal nonpregnant range.

### Prognostic Versus Therapeutic Use of the 2 g/L Threshold

As a prognostic value, a concentration at or below 2 g/L is well supported by observational data. Charbit et al. reported 100% positive predictive value for severe PPH at this concentration, in women already receiving second-line uterotonics [3]. But this describes a measured value correlating with an outcome that, in most cases, had already begun before the sample was drawn. Whether 2 g/L should also function as a therapeutic target, a concentration below which giving fibrinogen is expected to alter the clinical course, is separate and considerably more contested.

The case for retaining 2 g/L as a therapeutic action threshold is pragmatic, not mechanistic; it is the WHO’s stated therapeutic goal for active PPH [17]. Most relevantly here, it gives a Tier 2 team without viscoelastic testing a single actionable value. Clauss fibrinogen, where available, supplies that value directly, without interpretation against a platform-specific algorithm. Japan’s national clinical practice guide for massive PPH instead sets the transfusion-relevant threshold at fibrinogen below 1.5 g/L, alongside a shock index of 1.5, which is higher than the operational triggers used elsewhere [75].

Set against this, four considerations argue against treating 2 g/L as a validated, universal, or fixed causal threshold. First, the randomized trials enrolled almost no women with a documented concentration below 2 g/L. Absence of positive trial evidence at low concentrations is not evidence that treatment fails there.

Second, a single static value does not capture the rate at which fibrinogen is falling and rate matters as much as the value itself. Consider a woman whose fibrinogen has fallen rapidly from a third-trimester baseline of 6 g/L to 2.2 g/L. This may be more urgent than a stable antenatal value of 2.3 g/L.

Third, the same concentration carries different clinical meaning by cause. In atony-dominant hemorrhage with an identifiable, treatable mechanical source, 2 g/L may reflect straightforward dilution that corrects once bleeding is controlled. In evolving DIC from abruption or amniotic fluid embolism, the same value may be an early sign of a rapidly progressive consumptive process. Treating both identically, because they share a cut-off, disregards etiology.

Fourth, the threshold cannot distinguish a value produced by resuscitation from one reflecting the underlying disease process. Dilution from crystalloid or red cells can itself lower a measured concentration. A sample drawn before resuscitation may understate a deficit that appears only once dilution occurs.

In Tier 2 settings without viscoelastic testing, 2 g/L is the default action threshold. It is chosen for consistency with the WHO goal, and because no better locally validated alternative exists. In Tier 3 settings, a locally validated viscoelastic threshold is preferred once established because it captures the functional state more directly. RCOG’s guideline states that the appropriate fibrinogen intervention trigger is unknown. Its recommendation to maintain fibrinogen above 2 g/L reflects a pragmatic view of the evidence, not a validated target [15], and it identifies FIB-PPH as uninformative for the same reason given above [76,77,78,79].

## 8. Laboratory Assessment

### 8.1. Clauss Fibrinogen, Clot Quality, and Standard Laboratory Tests

Clauss fibrinogen remains the standard functional assay in most obstetric pathways and the test behind most thresholds. Two limitations matter in an emergency. The first is turnaround time. A figure of approximately 60–90 min has been reported from centers with an on-site coagulation laboratory [9]. Actual turnaround depends on sample transport distance, staffing, operating hours, whether an emergency channel exists, and local infrastructure. It can run substantially longer where samples are transported off-site, or substantially shorter with a dedicated near-patient laboratory. Nevertheless, the general limitation is consistent: a delay between sampling and availability of the result is typical when Clauss testing requires sample transport or batching. This is why early serial sampling, not the specific figure, is the transferable recommendation. The second limitation is measurement variability. Values vary with assay method, reagent, calibration, analyzer, heparin effect, and degradation products, and interlaboratory agreement is imperfect. A result should therefore be read against the local method and, where possible, a previous value from the same laboratory, rather than a universal cut-off [80].

Fibrin clot quality depends on fibrinogen concentration, molecular structure, thrombin concentration, factor XIII activity, platelet incorporation, pH, temperature, hematocrit, inflammatory modification, and fibrinolysis [48,49,51]. In acute obstetric hemorrhage, the problem may be quantitative, qualitative, or both: degradation products interfere with fibrin polymerization and generate a functional deficit that concentration alone does not capture. Factor XIII stabilizes the fibrin network through cross-linking; platelets provide mechanical support and bind fibrinogen via glycoprotein IIb/IIIa. Fibrinogen can therefore form a weak clot if factor XIII is depleted, platelets are low, or polymerization is impaired. PT, INR, and aPTT may stay near normal early and become abnormal only as multiple factors decline [3,4,5,6]. Platelet count, hemoglobin, blood gas, lactate, ionized calcium, temperature, and renal and hepatic function all provide complementary information. A normal early hemoglobin does not exclude major acute blood loss, and transfusion decisions should not rest on hemoglobin alone [17].

### 8.2. ROTEM, TEG, and Fibrin-Based Clot Firmness

ROTEM and TEG are distinct instruments, with different reagents, activators, and proprietary fibrin channel assays. Their results are not interchangeable: a threshold derived on one platform should not transfer to the other without independent validation.

TEG’s functional fibrinogen (FF, also reported as FLEV) assay inhibits platelets with abciximab. This does not fully block platelet contribution to clot strength in whole blood, and it has a measured clinical consequence. A prospective obstetric comparison enrolled 32 healthy volunteers, 34 healthy term pregnant women, and 32 women with PPH exceeding 1000 mL. TEG functional fibrinogen systematically overestimated Clauss in every group, by a bias of −159 mg/dL (approximately −1.6 g/L) in the hemorrhagic group, and correlated only moderately with Clauss. Unlike Clauss, it failed to distinguish healthy pregnant women from those with PPH (*p* = 0.186 versus *p* < 0.001 for Clauss). The authors concluded that TEG functional fibrinogen should not substitute for Clauss in this population [81].

Viscoelastic testing gives rapid information on clot initiation, development, firmness, and lysis. FIBTEM isolates the fibrin-based contribution to clot strength after platelet inhibition, with A5 amplitude available within about 10 min. FIBTEM A5 correlates with Clauss fibrinogen during PPH. But correlation strength, and the threshold that best identifies fibrinogen below 2 g/L, both vary across studies, devices, reagents, and cartridge versions (Table 4). Proposed intervention thresholds range from approximately 5–6 mm in an early single-center study to 15 mm in the OBS2 inclusion criterion. One UK center revised its own threshold from 12 mm to 8 mm [9,35,37,38,76,77,78,79].

Bell et al. found that the dual platelet-inhibited ROTEM FIBTEM assay correlated more strongly with Clauss than the single platelet-inhibited assay (r = 0.88 versus r = 0.63). A FIBTEM A5 of 7.8 mm on the dual-inhibited assay corresponded to the same Clauss concentration of 2 g/L [78]. A large multicenter Dutch validation cohort reached a similar conclusion. No single FIBTEM A5 cut-off performed well as a stand-alone test for Clauss fibrinogen ≤2 g/L. A locally Youden-optimal cut-off of 12 mm still misclassified 81% of women selected for treatment as having fibrinogen above 2 g/L [77]. A FIBTEM threshold should never be presented as a fixed, universal equivalent of a specific Clauss concentration [9,35,37,38,76,77,78].

Mallaiah et al. described introducing a ROTEM-guided algorithm for fibrinogen concentrate administration in major obstetric hemorrhage, reporting the feasibility of rapid point-of-care-guided replacement [38]. Where equipment and trained staff exist, such algorithms shorten the delay between recognizing coagulopathy and giving targeted treatment [37]. These assays accelerate interpretation, but they do not replace clinical assessment, source control, or the tests above, and they are not essential for effective first-response care.

## 9. Clinical Management Algorithm

Clinical evidence of coagulopathy denotes diffuse, non-surgical bleeding: oozing from venipuncture or intravenous sites, mucosal or gingival bleeding, hematuria, or failure of shed blood to clot in a no-additive tube at the bedside [79]. Empirical treatment under the major hemorrhage protocol should be activated when ongoing bleeding with hemodynamic instability—defined as a shock index at or above 1, a systolic blood pressure below 90 mmHg, or continued blood loss despite first-line uterotonics and tamponade—coincides with either clinical evidence of coagulopathy as defined above or a clinical context carrying a recognized high prior probability of coagulopathy: severe placental abruption, suspected amniotic fluid embolism, or intrauterine fetal death with prolonged retention.

Because no internationally validated algorithm exists for low-resource settings, the following tiered pathway is proposed as a pragmatic synthesis of current evidence and guideline principles.

### 9.1. Common Starting Sequence, All Tiers

1. Recognize PPH and measure blood loss objectively rather than by visual estimate.
2. Begin the first-response bundle simultaneously, not sequentially: uterine massage, a first-line uterotonic, intravenous access and tranexamic acid, fluids, and genital tract examination.
3. Identify and control the bleeding source in parallel with step 2.
4. If bleeding stops, continue surveillance and reassess venous thromboembolism risk once hemostasis is secure. If bleeding continues or progresses, proceed further.

### 9.2. Tier 1

1. No fibrinogen or viscoelastic result will become available at this tier; do not wait for one before proceeding.
2. Continue uterotonics and, where available, a non-pneumatic anti-shock garment, while activating referral in parallel. The WHO’s recommendation for uterine balloon tamponade is conditional on access to blood transfusion and surgical escalation if needed, both of which are absent by definition at this tier, so tamponade should not be treated as a routine Tier 1 substitute for prompt referral.
3. Refer immediately if the shock index reaches approximately 0.9–1.0 or blood loss approaches or exceeds 1000–1500 mL without response to first-line measures.
4. Temporary improvement after fluids or uterotonics does not cancel a transfer already triggered by step 3.

### 9.3. Tier 2

1. Send Clauss fibrinogen at presentation if bleeding continues, in parallel with source control and repeat serially rather than waiting for a single result.
2. If fibrinogen is below 2 g/L or clinical evidence of coagulopathy accompanies ongoing major bleeding, give cryoprecipitate, the preferred fibrinogen-specific product at this tier, dosed to local guidelines; reserve plasma for when cryoprecipitate is unavailable.
3. If severe abruption, suspected AFE, or overt DIC accompanies ongoing bleeding and no result is yet available, start empirical treatment under the major hemorrhage protocol while the sample is processed; draw the sample first wherever this will not delay treatment.
4. If bleeding is atony-dominant with no evidence of coagulopathy, prioritize source control, including uterine tamponade where indicated, and reserve blood product replacement.
5. Escalate or refer if bleeding continues despite these measures, or if surgical or critical care capacity is exceeded.

### 9.4. Tier 3

1. Use Clauss fibrinogen and/or the locally validated viscoelastic threshold to guide replacement.
2. If fibrinogen is below 2 g/L or FIBTEM is below the local threshold, give fibrinogen concentrate.
3. If the result is preserved, return to source control and uterine tamponade; randomized evidence does not support routine replacement.
4. If no result is yet available and severe abruption, suspected AFE, or overt DIC accompanies ongoing bleeding and clinical evidence of coagulopathy, apply the same empirical treatment criteria as Tier 2.
5. Escalate within the facility with surgical (uterine compression sutures, vessel ligation, uterine artery embolization, and hysterectomy) or critical care, rather than referring externally, unless a specific capacity is exceeded.

## 10. Management: Drugs, Doses, Targets, Monitoring, and Team Roles

### 10.1. Uterotonic Drugs and Doses

Intravenous oxytocin is first-line treatment: 10 IU diluted and given slowly, or 5–10 IU intramuscularly if intravenous access is not yet secured. An infusion of 40 IU in 500 mL isotonic crystalloid over 4 h follows if bleeding continues [17]. Oxytocin loses potency without a maintained cold chain; this is a specific constraint where refrigeration is unreliable [17].

The WHO’s 2025 consolidated guideline removed ergometrine/methylergometrine and the fixed-dose oxytocin–ergometrine combination from its prevention recommendations [17]. For treatment, the WHO still lists intravenous ergometrine, the fixed-dose combination, or a prostaglandin, including sublingual misoprostol 800 mcg, when oxytocin is unavailable or ineffective [17]. A retrospective cohort of 2,233,630 US delivery admissions linked methylergonovine exposure to acute coronary syndrome and myocardial infarction, which is rare in absolute terms, but consistent with a genuine drug effect [82,83]. Ergometrine and methylergometrine are therefore contraindicated outright in hypertensive disorders, including preeclampsia.

In many countries, misoprostol is registered only for gastric ulcer prevention. Its obstetric use, for both prevention and treatment of PPH, is off-label [84].

### 10.2. Multidisciplinary Team Activation and Responsibilities

Once PPH is diagnosed, roles should be pre-assigned rather than negotiated during the emergency. The birth attendant quantifies blood loss and starts the first-response bundle. The obstetrician controls the bleeding source. Anesthesia leads vascular access, resuscitation, and transfusion. A designated leader coordinates communication and triggers escalation [17,41]. At Tier 1, one person may hold several roles, which makes assigning escalation criteria in advance more important.

### 10.3. Tranexamic Acid

Tranexamic acid preserves blood clots by inhibiting plasminogen binding to fibrin, protecting both the formed clot and circulating fibrinogen. The WOMAN trial randomized 20,060 women with clinically diagnosed PPH across 21 countries, including substantial low- and middle-income representation. Tranexamic acid reduced death due to bleeding from 1.9% to 1.5% (risk ratio 0.81, 95% CI 0.65–1.00). The effect was greater within 3 h of birth, 1.7% versus 1.2% (risk ratio 0.69, 95% CI 0.52–0.91), and absent beyond 3 h [2]. Women were enrolled on clinical diagnosis, without a uniform minimum blood loss threshold, so severity was heterogeneous. Still, the mortality benefit supports early administration once PPH is diagnosed. This is one of the few interventions here with large, multinational trial evidence directly including low-resource settings.

The regimen is 1 g (100 mg/mL) intravenously at 1 mL/min, over approximately 10 min, as soon as PPH is diagnosed and within 3 h of birth. A second 1 g dose follows if bleeding continues after 30 min or restarts within 24 h. Faster administration can cause hypotension [2,17]. Tranexamic acid does not replace fibrinogen. It is recommended for clinically diagnosed PPH regardless of source, unless contraindicated [2,36].

### 10.4. Mechanical and Surgical Control

Hemostatic drugs cannot compensate for an uncontrolled source. Intrauterine balloon tamponade (pooled success 85.9% across 91 studies and 4729 women, lower in placenta accreta spectrum at 66.7% [85]) suits atony after other causes are reasonably excluded. The WHO’s own recommendation conditions its use on access to blood transfusion and surgical escalation if needed, which corresponds to Tier 2/3. Compression sutures, stepwise devascularization, arterial ligation, and hysterectomy follow a defined escalation when tamponade fails or is not the appropriate first step [17].

### 10.5. Fibrinogen Replacement: Indications, Products, Doses, and Targets

Fibrinogen concentrate is standardized and given in low volume without crossmatching. Viral inactivation method, storage temperature, and reconstitution time depend on the licensed product. These should be confirmed against the local label and not assumed to be uniform. A typical initial adult dose in major obstetric hemorrhage is 3–4 g (Table 5). Without continuing consumption, approximately 60 mg/kg raises plasma fibrinogen by approximately 1 g/L, which is a population estimate given directly in the RCOG guideline [15]. Reconstituted concentrate typically provides approximately 1 g of fibrinogen per 50 mL for common products.

Cryoprecipitate is more widely available, and remains the product specified by several national guidelines. It supplies fibrinogen together with factor VIII, factor XIII, the von Willebrand factor, and fibronectin. Its fibrinogen content and volume vary by jurisdiction, donor unit, pool size, and manufacturing method. The UK specification sets a minimum of 140 mg per unit; the US specification sets approximately 150 mg per unit [86]. The number of units or pools needed to deliver 3–4 g must therefore be calculated from the local specification. Strict ABO matching is not universally required. Fresh frozen plasma contains only approximately 2–3 g/L of fibrinogen, so 50 mL supplies about 100–150 mg. Correcting an isolated fibrinogen deficit with plasma therefore requires volumes that may themselves worsen dilution. Plasma at 15–20 mL/kg suits global factor deficiency but is inefficient for isolated fibrinogen replacement [39,40,41,42,43,44]. Individual components remain preferred when available [17,41].

In the FIBRES trial, 735 adults bleeding after cardiopulmonary bypass received fibrinogen concentrate (4 g) or cryoprecipitate (10 units). Allogeneic transfusion for 24 h did not differ (ratio 0.96), establishing non-inferiority of concentrate, not superiority of either product [87]. A 2025 systematic review pooling this and three further trials (945 patients) found no consistent difference in transfusion outcomes [88]. A single-center trial randomized 88 adults with post-bypass hypofibrinogenemia to cryoprecipitate (*n* = 40) or concentrate (n = 48). Cryoprecipitate produced a numerically larger 24 h fibrinogen increment, 125 versus 96 mg/dL, at lower direct cost [89]. A parallel cost-effectiveness analysis found fibrinogen concentrate cost-effective only after excluding a minority of critically ill outliers [90]. Cryoprecipitate, meanwhile, has been reported to be 3–4 times cheaper than concentrate per gram of fibrinogen delivered, in US institutional costing [91].

**Table 5:** Practical comparison of fibrinogen replacement products.

Product	Fibrinogen Content	Storage and Preparation	Relative Cost	Transfusion-Related Risk	Low-Resource Suitability
Fibrinogen concentrate	Standardized per lot; ~1 g/50 mL reconstituted	Lyophilized, room temperature, ~3 y shelf life; reconstitute ~15 min, use within 8 h [91,92]	Highest per gram; ~3–4 × cryoprecipitate [90,91]	Viral-inactivated pharmaceutical; lowest infection transmission risk; allergic reactions reported	Room-temperature storage; cost and registration are the limiting factors
Cryoprecipitate	Variable by jurisdiction and donor: UK minimum 140 mg/unit, US ~150 mg/unit; population range reported as wide as 3–30 g/L equivalent [86]	Frozen −18 to −25 °C, ~1 y; requires thawing (~20–30 min); conventional post-thaw shelf life 4–6 h [86]	Lower per gram than concentrate; ~3–4 × cheaper [91]	Pooled donor product; standard (non-pathogen-reduced) preparations are not virally inactivated; TRALI/allergic risk	Often already stocked for other indications; frozen cold chain and thawing capacity are the binding constraint
Fresh frozen plasma	Low, ~2–3 g/L; 50 mL supplies only ~100–150 mg	Frozen, comparable to cryoprecipitate; thawing required	Lowest acquisition cost per unit; least efficient per gram fibrinogen delivered	Same donor-derived risks as cryoprecipitate; largest infused volume raises dilution/overload risk	Most widely available component; reasonable for multi-factor deficiency, inefficient as a dedicated fibrinogen source

Abbreviation: TRALI, transfusion-related acute lung injury.

### 10.6. Monitoring and Surveillance

Surveillance continues after apparent control, since recurrent atony, retained tissue, and delayed coagulopathy all occur. Uterine tone, vital signs, and laboratory response should be reassessed. Thromboprophylaxis, once indicated, starts only after hemostasis is secure and coagulopathy corrected, following local obstetric guidance [41]. Women who received fibrinogen replacement, particularly those with a known fibrinogen disorder, require explicit thrombotic risk assessment, rather than an assumption that recent hypofibrinogenemia excludes thrombotic risk [61,62,63,64,68].

## 11. Clinical Implications in Limited Resources

Correcting antenatal anemia reduces the physiological deficit tolerated before fibrinogen-relevant thresholds are reached. It is a core element of patient blood management in obstetrics [41]. Antenatal and admission assessment should flag anemia, placenta previa or accreta spectrum, prior PPH, coagulation disorders, severe preeclampsia, suspected abruption, and multiple pregnancy. Many PPH cases occur without a known risk factor [17,41]. Active management of the third stage of labor reduces incidence [17,93,94]. Facilities should verify blood group and antibody information, define emergency release procedures, stock quality-assured uterotonics and tranexamic acid, maintain calibrated drapes, and prepare a hemorrhage cart with a written escalation pathway [17].

## 12. Congenital Fibrinogen Disorders in Pregnancy

Congenital fibrinogen disorders are rare, but pregnancy’s hemostatic demands can unmask them as miscarriage, antepartum bleeding, PPH, or thrombosis. Quantitative disorders (afibrinogenemia, hypofibrinogenemia) and qualitative disorders (dysfibrinogenemia) arise from FGA/FGB/FGG variants. They are not purely hemorrhagic: reduced or dysfunctional fibrin also weakens platelet bridging and thrombin sequestration. This has been proposed to contribute to the thrombotic events reported in a substantial minority of patients, despite their bleeding tendency [45,46,47,48,49,50,51,61,62,63,64]. Heritable dysfibrinogenemia is estimated at approximately 15 per 100,000 among individuals tested. However, it is probably underestimated, since many carriers remain asymptomatic [47,48,49,50,51].

The obstetric outcome literature is small and predominantly European. The Francophone-led international Fibrinogest study (425 pregnancies) reported PPH in 19.9% [65]. The Prospective Rare Bleeding Disorders Database (59 women) reported miscarriage in 23% and PPH in 36% [66]. Reported treatment targets vary by genotype and by bleeding or thrombotic history, rather than converging on one value. The 2024 ISTH SSC communication accordingly recommends individualized, specialist-set trough targets, not a universal concentration [68]. Inherited disease nevertheless explains only a small minority of low fibrinogen in pregnancy overall. Fewer than 5% of the 124 women identified with fibrinogen below 2.0 g/L in the largest population-based series had a pre-existing inherited disorder [19]. Routine antigen testing or genetic sequencing is therefore not justified in unselected PPH.

## 13. Research Priorities for Low-Resource Settings

Bedside clotting observation needs validation against Clauss fibrinogen for sensitivity and reproducibility, and a Clauss measurement at diagnosis needs testing against clinical assessment for its effect on triage and outcome. Blood loss and shock index thresholds for empirical replacement need defined operating characteristics where testing is unavailable. Cold chain-free point-of-care assays need validation and costing analysis. The optimal product remains unresolved where concentrate is unavailable, as do fibrinogen trajectories in high-anemia populations.

Other priorities need reference laboratory or multicenter collaboration. They include mechanistic characterization of obstetric coagulopathy across the coagulation–fibrinolysis pathway. They include validation of pregnancy-adapted DIC scoring, since nonpregnant criteria do not transfer. They include genetic investigation, including exome sequencing, where recurrent loss, unexplained PPH, or discordant Clauss–antigen results suggest inherited disease. And they include registries across diverse populations, including low- and middle-income settings, to define postpartum thrombotic risk after replacement.

## 14. Limitations

This review has several limitations. Study selection was performed by the author team without a pre-registered protocol, as in a systematic review. The primary studies cited use heterogeneous PPH definitions, timing conventions, and blood loss measurement methods, which limits direct numeric comparison across studies. Fibrinogen assays are themselves heterogeneous, and the timing of fibrinogen measurement relative to bleeding onset differs substantially across the studies cited. The evidence base is predominantly generated in high-resource healthcare systems. Randomized-trial evidence on fibrinogen replacement is limited specifically among women with severe hypofibrinogenemia.

The 2 g/L threshold used throughout this review, and by several guidelines, carries uncertainty. It functions as a practical, guideline-endorsed action point rather than a biologically validated cut-off. The true concentration–outcome relationship remains incompletely characterized.

## 15. Conclusions

Pregnancy-associated fibrinogen deficiency is unusual. Fibrinogen is normally elevated in pregnancy, while qualitative dysfunction may be present despite a near-normal concentration. During active bleeding, a low or rapidly falling fibrinogen concentration is strongly associated with hemorrhage severity. Interpretation depends on cause, pregnancy baseline, timing, resuscitation, and assay method. The same numeric concentration carries different meanings in normal late pregnancy, atony-dominant PPH, placental abruption, amniotic fluid embolism, sepsis-associated DIC, massive transfusion, and congenital fibrinogen disease.

During active PPH, fibrinogen below 2 g/L remains a major warning signal and a practical, guideline-endorsed treatment threshold. No completed randomized trial has demonstrated a clinical benefit from fibrinogen replacement specifically in women with severe hypofibrinogenemia. Values above this level may still be abnormal when they represent a rapid fall from pregnancy baseline or occur in placental-source hemorrhage.

## Figures and Tables

**Table 3:** Randomized controlled trials of fibrinogen replacement in postpartum hemorrhage.

Trial	Population, Inclusion, and N Randomized	Blood Loss and Baseline Fibrinogen at Inclusion	Intervention	Primary Outcome	Conclusion
FIB-PPH (Wikkelso et al., 2015) [7]	PPH ≥ 500 mL vaginal or ≥1000 mL cesarean, pre-emptive design; N = 249	Mean blood loss 1459 mL; mean fibrinogen 4.5 g/L	Fixed 2 g fibrinogen concentrate vs. placebo	Red cell transfusion to 6 weeks postpartum	No reduction in transfusion or secondary outcomes
FIDEL (Ducloy-Bouthors et al., 2021) [8]	Persistent PPH after vaginal delivery, escalation from oxytocin to prostaglandin; N = 437	Mean blood loss 877 mL; mean fibrinogen 4.1 g/L	Fixed 3 g fibrinogen concentrate vs. placebo	Composite outcome (transfusion/invasive procedure)	No improvement in composite outcome; hypofibrinogenemia reduced
OBS2 (Collins et al., 2017) [9]	PPH 1000–1500 mL, screened by FIBTEM A5 ≤ 15 mm with continued bleeding; 55 of 663 screened randomized, only 7 had fibrinogen ≤ 2 g/L	Screening range 1000–1500 mL; selected for FIBTEM A5 ≤ 15 mm	Weight-adjusted fibrinogen concentrate vs. placebo	Allogeneic units transfused	Adjusted IRR 0.72 (95% CI 0.3–1.7), not significant; no benefit predicted above FIBTEM A5 12 mm or fibrinogen 2 g/L
Jokinen et al., 2023 (pilot) [72]	PPH > 1500 mL; 60 randomized, 54 analyzed	Not fixed at entry; not selected by fibrinogen	ROTEM-guided treatment algorithm vs. conventional care	Red cell units transfused	2 vs. 3 units, *p* = 0.399 (not significant); lower plasma use and total blood loss in ROTEM-guided group
ACROBAT (Green et al., 2022, pilot) [73]	Severe PPH, active bleeding <24 h of birth requiring ≥1 RBC unit; cluster-randomized, not selected by fibrinogen	Not fixed at entry; not selected by fibrinogen	Early cryoprecipitate vs. standard care	Feasibility; RBC units at 24 h (secondary)	RBC transfusion, surgery and ICU admission numerically lower with cryoprecipitate; not powered for efficacy

**Table 4:** FIBTEM A5 thresholds proposed for identifying Clauss fibrinogen ≤ 2 g/L in postpartum hemorrhage by study, platform, and population.

Study/Algorithm	Platform and Population	Proposed FIBTEM A5 Threshold	What It Correlates with	Limitations
Huissoud et al., 2009 [76]	Single French center; ROTEM; PPH	Approximately 5–6 mm	Fibrinogen below approximately 1.5–2 g/L	Small, single-center early study; one of the first proposed obstetric cut-offs
Collins et al., 2014 [6]	Cardiff, UK; ROTEM; prospective cohort of 356 women with PPH 1000–1500 mL	Below 10 mm	Positive predictive value of 71% for progression to any transfusion (versus 75% for Clauss fibrinogen <2 g/L)	Predictive performance similar to, not better than, Clauss fibrinogen in this cohort
Mallaiah et al., 2015 [38]	Liverpool, UK; ROTEM Delta, single platelet-inhibited; major obstetric hemorrhage	≤12 mm	Local intervention threshold for a clinical treatment algorithm	Introduced as a treatment decision point, not validated as a stand-alone diagnostic cut-off
OBS2/Collins et al., 2017 [9]	Multicenter UK RCT; ROTEM; PPH 1000–1500 mL	≤15 mm (inclusion criterion)	Approximately fibrinogen <3 g/L; pre-specified analysis suggested no benefit above 12 mm	Only 55 of 663 screened women met the randomization criterion; only 7 had fibrinogen ≤2 g/L
Gillissen et al., 2023 (TeMpOH-2, preprint) [77]	Netherlands, 3 centers; ROTEM Delta and Sigma; 511 women, 637 samples	Locally Youden-optimal at 12 mm; no cut-off performed well overall	Sensitivity 87%, specificity 81% at 12 mm; 81% of women selected at this threshold had fibrinogen >2 g/L	Correlation differed materially by device (Delta r = 0.63 vs. Sigma r = 0.76); authors conclude no single cut-off is reliable as a stand-alone test
OBS Cymru/All Wales Guideline, 2023 [79]	Wales, national program; ROTEM Sigma after reagent/cartridge update	Revised to ≤8 mm	Same clinical intervention point as the prior 12 mm threshold, under a new reagent	Threshold changed with platform/reagent, not with any change in the underlying clinical question
Bell et al., 2024 [78]	Cardiff, UK; ROTEM Sigma, dual- vs. single-platelet-inhibited assay	7.8 mm (dual-inhibited) vs. 11 mm (single-inhibited)	Both correspond to the same Clauss fibrinogen, 2 g/L	Directly demonstrates that a numerically identical fibrinogen concentration maps to different FIBTEM thresholds depending on assay configuration

Note: The values listed are platform-, reagent- and algorithm-specific decision points derived in the cohorts shown. They are not interchangeable between ROTEM and TEG or between single- and dual-platelet-inhibited assays, and none should be read as a universal equivalent of a given Clauss fibrinogen concentration. Abbreviations: FIBTEM, fibrin-based rotational thromboelastometry assay; PPH, postpartum hemorrhage; and RCT, randomized controlled trial.

## Data Availability

No new data were created or analyzed in this study. Data sharing is not applicable to this article.
